# Kinase Inhibitors as Potential Therapeutic Agents in the Treatment of COVID-19

**DOI:** 10.3389/fphar.2022.806568

**Published:** 2022-04-04

**Authors:** Rajashri R. Naik, Ashok K. Shakya, Safwan M. Aladwan, Mohamed El-Tanani

**Affiliations:** ^1^ Department of Biopharmaceutics and Clinical Pharmacy, Al-Ahliyya Amman University, Faculty of Pharmacy, Amman, Jordan; ^2^ Faculty of Allied Medical Sciences, Al-Ahliyya Amman University, Amman, Jordan; ^3^ Faculty of Pharmacy, Pharmacological and Diagnostic Research Centre, Al-Ahliyya Amman University, Amman, Jordan; ^4^ Department of Pharmaceutical Sciences, Faculty of Pharmacy, Al-Ahliyya Amman University, Amman, Jordan

**Keywords:** tyrosine kinase inhibitor, repurposing agents, ABL and SRC kinase inhibitors, NAK and AXL kinase inhibitor, EGFR inhibitiors, CDK inhibitors, COVID-19

## Abstract

Corona virus is quickly spreading around the world. The goal of viral management is to disrupt the virus’s life cycle, minimize lung damage, and alleviate severe symptoms. Numerous strategies have been used, including repurposing existing antivirals or drugs used in previous viral outbreaks. One such strategy is to repurpose FDA-approved kinase inhibitors that are potential chemotherapeutic agents and have demonstrated antiviral activity against a variety of viruses, including MERS, SARS-CoV-1, and others, by inhibiting the viral life cycle and the inflammatory response associated with COVID-19. The purpose of this article is to identify licensed kinase inhibitors that have the ability to reduce the virus’s life cycle, from entrance through viral propagation from cell to cell. Several of these inhibitors, including imatinib, ruxolitinib, silmitasertib, and tofacitinib (alone and in conjunction with hydroxychloroquine), are now undergoing clinical studies to determine their efficacy as a possible treatment drug. The FDA approved baricitinib (a Janus kinase inhibitor) in combination with remdesivir for the treatment of COVID-19 patients receiving hospital care in November 2020. While *in vitro* trials with gilteritinib, fedratinib, and osimertinib are encouraging, further research is necessary before these inhibitors may be used to treat COVID-19 patients.

## Introduction

Corona virus emerged in late December 2019 in an uninfected person in Wuhan, China, and subsequently spread exponentially over the world because of its quick human transmission ability (Lai et al., 2020; Zhu et al., 2020). As of 29th October 2021, the corona virus has infected around 245.37 million people and killed approximately 4.97 million. Doctors, pharmaceutical corporations, and university scientists are all working hard to create many vaccines that have been approved to stop the disease from spreading (WHO, 2021b). In October 2020, the FDA approved remdesivir for COVID–19 individuals aged 12 and older who required hospitalization. The WHO does not recommend remdesivir for COVID–19 patients, regardless of their severity, due to a lack of evidence that it increases survival rates in hospitalized patients. The WHO advises treating COVID–19 patients with corticosteroids for 7–10 days, but not otherwise healthy persons. The FDA has approved the use of antibodies such as bamlanivimab and etesevimab in patients with mild to severe COVID–19 only in emergency (FDA-News, 2020b). According to the findings, monoclonal antibodies should not be used in hospitalized or mechanically ventilated patients. As a result, therapeutic options are limited, and experts fear the virus will remain longer than previously imagined.

To develop potential antiviral medicines, one must first understand the corona virus’s basic features and life cycle. The corona virus is enveloped and has the largest viral RNA genome, ranging between 26 and 32 kilobases (Pillaiyar et al., 2016; Pillaiyar et al., 2021). It is a positive RNA virus. Currently, seven human corona viruses have been identified and classified into four coronaviridae genera (Pillaiyar et al., 2020). HCoV-229E and HCoVOC43 are the two α-CoVs. β-CoVs include the HCoV-HKU1, HCoV-NL63, SARS-CoV-1, MERS-CoV, and SARS-CoV-2 (Yin and Wunderink, 2018; Guo et al., 2020). These viruses are typically nosocomial, whereas SARS-CoV-2 is human-to-human. SARS-CoV-1 appeared in 2002–2003, infecting 8,096 individuals and killing 10%. (WHO, 2015; Pillaiyar et al., 2016). MERS-CoV was discovered in 2012 and infected 2,578 people, killing 888. (Pillaiyar et al., 2015; Anthony et al., 2017; WHO, 2021a). SARS-CoV–2 is a Betacoronavirus that shares 80% of its genes with SARS–CoV and 20% with MERS–CoV (Baltimore, 1971; Lu et al., 2020) The origin, genetic makeup, and clinical symptoms of the disorders are all comparable (Katsiki et al., 2020).

The coronavirus life cycle has four stages: entrance, replication, assembly, and release. I proteases essential for viral protein proteolytic cleavage and replication, such as M^pro^ and the papain-like protease PL^pro^, must be present (Sungnak et al., 2020). Important steps in the virus’s life cycle. Treatment intervention at any of these stages may help coronavirus-related diseases (McKee et al., 2020).

COVID-19 infection presents immediately, while between 25 and 50% of patients are asymptomatic. The SARS-CoV-2 virus causes symptoms such as dry cough, fever, fatigue, sore throat, and shortness of breath (Yuki et al., 2020; Attaway et al., 2021). Among extreme cases, the virus-induced hyperinflammatory response can produce ARDS (acute respiratory distress syndrome), which is one of the leading reasons of mortality in COVID–19 patients. Pro-inflammatory cytokines cause a cytokine storm in ARDS. IL-2, IL-6, IL-7, GM-CSF, TNF-α, IFN-inducible protein 10, MIP-1α, and MCP-1 are only a few of the cytokines (MCP-1). As a result, Bouhaddou et al. (2020) report an increase in mortality due to ARDS, respiratory failure, and multi-organ failure (Xu et al., 2020; Zhu et al., 2020). Long et al. found that asymptomatic SARS-CoV-2 infections had lower virus-specific IgG levels (*p* = 0.005) than symptomatic SARS-CoV-2 infections (median S/CO, 3.4; interquartile range, 1.6–10.7). Asymptomatic people had lower levels of numerous pro- and anti-inflammatory cytokines. These data imply that asymptomatic persons had a weaker immune response to SARS-CoV-2 infection (Long et al., 2020).

All stages of the virus life cycle consume host resources. Cellular kinase appears to be essential for life. These kinases are suspected of being involved in the transmission of SARS-CoV-2. These kinases are also linked to pneumonia-like symptoms, inflammation, and fibrosis in corona virus infections (Bouhaddou et al., 2020; Galimberti et al., 2020; Weisberg et al., 2020). This includes cytokine synthesis and response, as well as the protein linked with inflammation (TGF-β) (Schindler et al., 2007; Bachstetter and Van Eldik, 2010). Thus, kinase inhibitors may be antiviral, anti-inflammatory, anti-cytokine, and anti-fibrotic, making them useful in the battle against infection or pandemic. Protein kinases are now being targeted by drugs. Protein kinases are targets for about 25-30% of novel medications generated by pharmaceutical companies. Many promising kinase inhibitors may be beneficial in treating COVID-19’s severe and occasionally deadly symptoms. Infection can be prevented by directly targeting the virus and reducing clinical signs using kinase inhibitors. Kinases can also be utilized to boost the efficacy of other antiviral medicines or tailored treatments for SARS-CoV-2. Many kinase inhibitors are now being tested in clinical studies to see if they work as a viable cure.

There is limited study on kinase inhibitors as possible antiviral and chemotherapeutic agents, but less on their utility against COVID-19. This article highlights COVID-19 kinases and their inhibitors that reduce symptoms and enhance patient outcomes. This page also includes FDA-approved kinase inhibitors now in clinical trials (Supplementary Table S1), gives an overview of the clinical trials that are underway, completed or terminated.

### Protein Kinase Inhibitors as a Possible Antiviral Agent

The repurposed medications are only used to treat the symptoms of respiratory illnesses like SAR-CoV, MERS-CoV, and SARS-CoV-2. Many of these FDA approved medications are examined for tolerance and toxicity as well as reducing illness symptoms. Many proteins, including host cell proteins, are targets of kinase inhibitors (FDA approved). These qualities can be employed for antiviral therapy. These medications can be repurposed and made available to patients, reducing the time, effort, and money required to produce new treatments.

**FIGURE 1:**
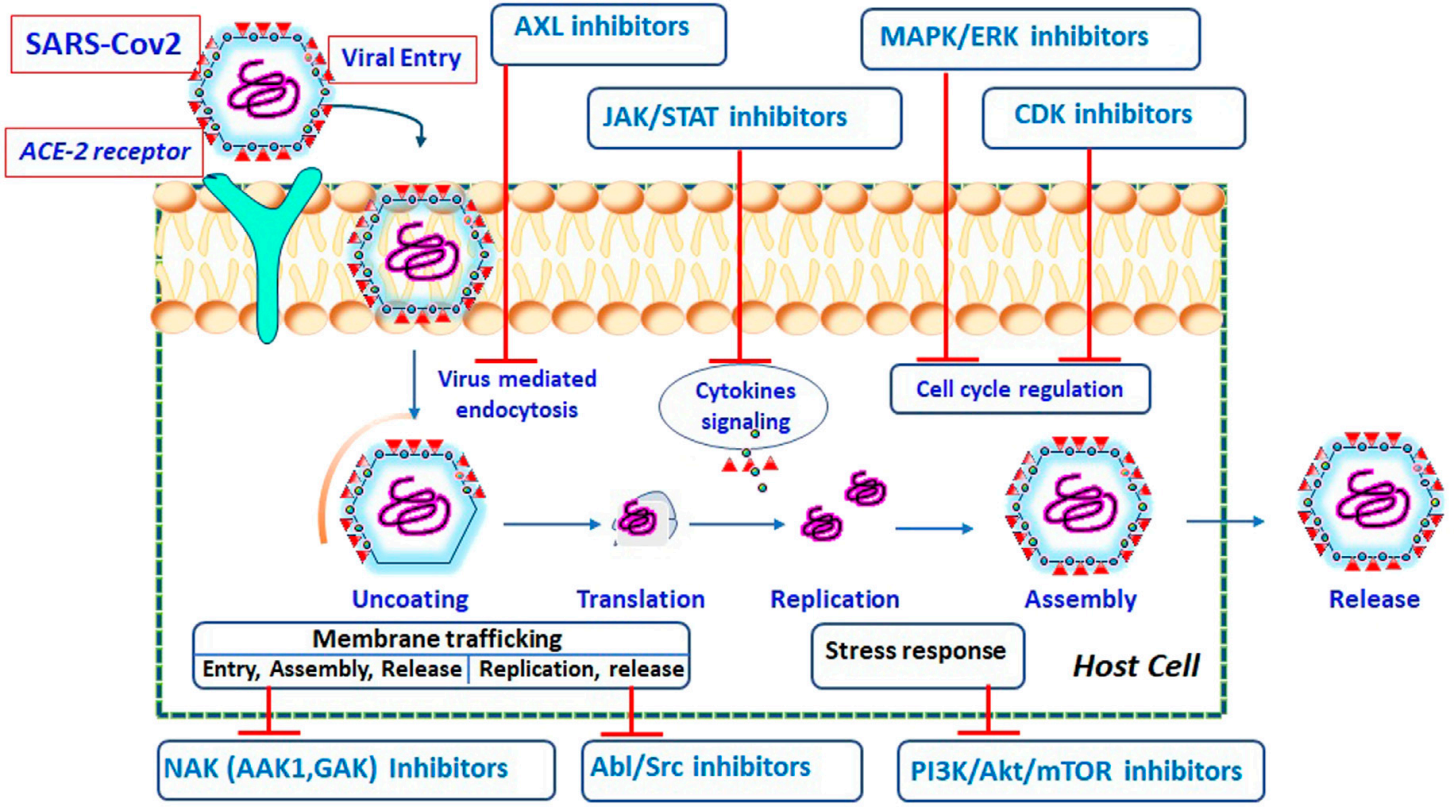
Schematic representation of SARS-CoV-2 life cycle at different stages and possible inhibitors acting on specific target (Pillaiyar and Laufer, 2021).

The corona virus life cycle (from entrance to release) is schematically depicted in Figure 1, along with the potential kinase inhibitors engaged at various stages of the viral cycle.

### Inhibitors of ABL and SRC Kinases

Signaling kinases like ABL kinases are involved in cell proliferation, adhesion, and stress response. According to various experimental research, ABL kinase has been implicated in several stages of the virus life cycle in viruses such as the Ebola virus (García et al., 2012; Kouznetsova et al., 2014), coxsackievirus (Coyne and Bergelson, 2006), and vaccinia virus (Newsome et al., 2006). ABL kinases are involved in the entry and replication of the virus and have been recently identified as a possible target for coronavirus infection. Dyall and his coworkers screened approved or experimental drugs as possible therapeutic antiviral agents for SARS-CoV-1 and MERS-CoV (Dyall et al., 2014). As a result of that, three signaling kinase inhibitors, imatinib, dasatinib, and nilotinib, were developed. Imatinib and dasatinib inhibit replication in SARS-CoV-1 and MERS-CoV, while nilotinib inhibits replication in SARS-CoV-1 only. SARS-CoV-1 was sensitive to all of these inhibitors. According to the investigation, imatinib suppressed viral replication by preventing the fusion of the coronavirus virion to the endosomal membrane during the early stages of the viral life cycle (Coleman et al., 2016; Sisk et al., 2018).

Both imatinib and dasatinib are orally available ABL kinase inhibitors. Imatinib is a protein kinase inhibitor used in the treatment of chronic myeloid leukemia (CML) (Kalmanti et al., 2015), whereas dasatinib is the second-generation ABL kinase inhibitor used in the treatment of CML and acute lymphoblastic leukemia (ALL) (O'Hare et al., 2005). Nilotinib inhibits replication of SARS-CoV-1 in micromolar concentrations but has had no significant inhibitory activity against MERS-CoV with 39% inhibition. Nilotinib in ABL-expressing cells blocks ABL kinase activity and proliferation more effectively than imatinib (Manley et al., 2005; Olivieri and Manzione, 2007; Breccia and Alimena, 2010; Jabbour et al., 2010). The mechanistic analyses revealed that SARS-CoV-1 and MERS-CoV used ABL2 kinase to enter the host rather than ABL1. Imatinib, which inhibits the ABL2 kinase, prevents the virus from entering the body.

In ABL2 kinase, the SH2 and SH3 domains hold the proteins in a dormant state (Colicelli, 2010). Binding of the substrate to either the SH2 or SH3 domain may turn ABL2 from an inactive form to its active form (Bradley and Koleske, 2009). Binding of the ATP to the catalytic site allows the kinase activity by conformation of the ABLs into their active form. It has been suggested that the infection of coronavirus increases the active conformation of the ABL2 kinase through substrate binding and phosphorylation downstream (Tokarski et al., 2006). Imatinib inhibits the ABL2 kinase activity by blocking the phosphorylation of proteins in cells.

As mentioned earlier SARS-CoV-1 and SARS-CoV-2 have around 80% similarity in their genome sequence. It is assumed that imatinib is supposed to inhibit SARS-CoV-2 also. Zhao and his colleagues studied the anti SARS-CoV-2 activity of imatinib using pseudo-type viruses with COVID-19 spike proteins in Caco-2 cells (Zhao et al., 2020). Imatinib at concentrations up to 10 μM did not affect the entry or infection of SARS-CoV-2. This supports the results of Zhao et al. that suggested the weak inhibitory activity of imatinib in inhibiting the replication of SARS-CoV-1 in vero cells with EC50 9.85 μM (Dyall et al., 2014).

The production of pro-inflammatory cytokines causes a cytokine storm, which is a hallmark of ARDS in severe COVID-19. Due to its immunomodulatory effects, imatinib inhibits the cytokine signaling receptors (PDGFR, c-Kit, and CSF1R), resulting in the suppression of a cytokine storm (Mohty et al., 2005; Zhao et al., 2020). Therefore, imatinib may have therapeutic benefits in the inhibition of COVID-19 infection. In a recently published preprint, imatinib was found to suppress the SARS-CoV-2 replication with an IC_50_ value of 130 nM (Mulgaonkar et al., 2020), which was in contrast to the Zhao studies (Zhao et al., 2020). Imatinib binds to the receptor-binding domain (RBD) of the spike proteins of SARS-CoV-2 at a concentration of 2.32 M, which indicates that there was no interaction specifically between SARS-CoV-2 RBD and ACE2 receptors. As a result, imatinib inhibited cellular tyrosine kinase as well as viral fusion, blocking virus replication. Three clinical trials are currently underway in different parts of the world to investigate the clinical efficacy of imatinib on COVID-19 patients: NCT04394416 (United States), EudraCT 2020-001236-10 (Netherlands), and NCT04357613 (France).

It should be emphasized that tyrosine kinase inhibitors (TKI) are critical in the prevention of SARS-CoV-2 infection in patients with Ph + ALL and CML (Galimberti et al., 2020). Galimberti et al. (2020) stated that in an Italian cohort analysis of Ph + ALL and CML patients, they discovered that just a few individuals have COVID-19. They concluded that the usage of TKIs may have contributed to the reduced rate of infection. It is fascinating that patients treated with TKIs such as imatinib or nilotinib exhibited increased expression of various pro-immune genes such as CD28 and IFN, but decreased expression of anti-immune genes such as ARG-1, CEACAMI, and FUT4. As discussed previously, SARS-CoV infection occurs through a series of steps: receptor engagement, conformational modification of S-glycoprotein, and cathepsin-L-induced proteolysis within endosomes (Simmons et al., 2005). It has been shown that the infection of SARS-CoV can be inhibited by targeting the inhibitors of cathepsin L (Simmons et al., 2005). In keeping with the preceding point, it should be noted that cells can be treated with cathepsin inhibitors as well as serine protease inhibitors to induce complete viral entry and replication. Camostat is a protease inhibitor that blocks the activity of type II trans-membrane serine protease (TTSP). Shirato et al. (2013) describe how TMPRSS2 cleaves the S glycoprotein of CoV and helps in the entry of the virus into the host cell.

It was observed that blocking cathepsin-L can prevent SARS-CoV-1 infection, and that a combination of EST (a cathepsin inhibitor) and camostat can accomplish total viral entrance and replication of SARS-CoV-1 (a protease inhibitor). Imatinib, a kinase inhibitor, has been shown to impact the function, localization, and activity of TMPRSS2 (Coleman et al., 2016).

Because SARS-CoV-2 employs SARS-CoV receptors (ACE2 and TMPRSS2 protease), this medication combination therapy may be promising and might be investigated further as a possible COVID-19 infection therapy. ABL and Arg kinases also cause endosomal cathepsin L release in cancer cells (Tripathi et al., 2018). Thus, testing ABL inhibitors’ capacity to block cathepsin L replication may yield promising findings, and it would be useful to test each of these targets for COVID-19 infection. Further research may be necessary to assess the therapeutic advantages of ABL inhibition in the COVID-19 infection.

### SRC Kinases

The SRC family of kinases (SFKs) consists of nine members: SRC, Yes, Fyn, Fgr, Lck, Hck, Blk, Lyn, and Yrk. These are non-receptor tyrosine kinases that play an important role in signal transduction. These members regulate a number of biological processes that promote cell survival and motility. SKFs also play a critical role in the viral life cycle (Pagano et al., 2013), and several of these SRC family kinases have been linked to SARC-CoV-2 replication. Shin and others revealed that saracatinib, an inhibitor of ABL/SRC kinases, suppresses the MERS-CoV life cycle at micromolar doses (Shin et al., 2018). According to their theory, saracatinib suppressed MERS-CoV through inhibiting the SFK signaling pathway. Later siRNA knock-down investigations revealed Lyn and Fyn as saracatinib targets that resulted in a reduction in MERS-CoV (Kumar et al., 2016; Shin et al., 2018), indicating that they are required for viral replication. Saracatinib also synergized with gemcitabine (a thymidylate synthase inhibitor), which indicated anti-MER-CoV efficacy (Singh et al., 2015). Additionally, antiviral activity was detected against the HCoV-229E and HCoV-OC43 viruses. SRC kinases may be targets for viral infection and replication in viruses such as HCV77 and dengue (Kumar et al., 2016). Dengue virus replication requires SRC kinase (Csk), a critical host kinase. When siRNA was used to knock down Csk expression, the RNA burden was controlled, and when the cell lacked SRC kinases (Fyn, SRC, and Yes), dengue infection was diminished (Kumar et al., 2016). This indicates that SRC kinases are required for dengue virus replication and assembly. Saracatinib and dasatinib inhibited the replication of Fyn RNA and were hence effective against dengue virus (de Wispelaere et al., 2013).

### Inhibitors of NAK (Numb—Associated Kinase) and AXL Kinase

The NAK family of kinases is required for the replication of a large number of viruses. They are comprised of four human homologues: I AK1 adaptor protein-associated kinase-1 (AAK1), 2) BMP-2-inducible kinase (BIKE/BMP2K), 3) cyclin G-associated kinase (GAK), and 4) myristoylated and palmitoylated serine/threonine kinase-1 (MPSK1). All of these homologues are significant, but adaptor-associated kinase (AAKI) and GAK are particularly so. AAKI increases endocytosis, whereas GAK modulates it. These two homologues are involved in the entrance, assembly, and dissemination of viruses such as dengue, Ebola, HCV, SARS-CoV-1, SARS-CoV-2, and MERS-CoV. (Conner and Schmid, 2002; Lee et al., 2005; Neveu et al., 2012; Neveu et al., 2015; Cao YC. et al., 2020). Numerous kinase inhibitors specifically target the AAKI and GAK kinases. Sunitinib, a multi-targeted kinase inhibitor licensed by the FDA, is used to treat renal cell carcinoma (RCC) and imatinib-resistant gastrointestinal stromal tumor (GIST) (Kelly et al., 2021). Erlotinib is an EGFR tyrosine kinase inhibitor that has been authorized by the Food and Drug Administration (FDA) for the treatment of metastatic non-small cell lung cancer (NSCLC) and pancreatic cancer.

Sunitinib and erlotinib inhibit the AAKI and GAK and display antiviral efficacy against dengue and Ebola viruses in cultured cells (Hirsch et al., 2005; Bekerman et al., 2017). They demonstrated antiviral action at the molecular level by blocking the intracellular trafficking of AAKI and GAK through AP (Bekerman et al., 2017). Sunitinib and erlotinib were demonstrated to reduce morbidity and death in dengue and Ebola virus-infected mice when used in combination. However, it should be noted that in COVID-19 patients, a substantially greater dose of sunitinib and erlotinib may be necessary to inhibit AAKI and GAK kinases (Richardson et al., 2020).

The FDA-approved Janus kinase (JAK) inhibitor baricitinib was recently presented as a prospective SARS-CoV-2 inhibitor and regarded as a possible option to treat COVID-19 infection by a research group from Benevolent Al and Imperial College London (Richardson et al., 2020).

They hypothesized that barcitinib prevented viral infection by inhibiting the AAKI kinase and binding to GAK. Barcinitib has been demonstrated to be effective in decreasing ARDS in patients with severe COVID-19. The FDA approved the use of baricitinib in combination with remdesivir to treat COVID-19 patients receiving hospital treatment in November 2020. (FDA-News, 2020a).

Baricitinib inhibited not only ARDS in COVID-19 patients, but also the migration and intracellular assembly of SARS-CoV-2. With a therapeutic dosage of 2 or 4 mg once a day, it is significantly superior to any other inhibitor as an antiviral drug. Baricitinib treatment improved the clinical outcomes of COVID-19 patients. Baricitinib is used to treat rheumatoid arthritis (RA) and has been shown to be successful in RA clinical studies. In individuals with rheumatoid arthritis, baricitinib had a high oral bioavailability and was well tolerated (Hoang et al., 2021). Due to its low plasma protein binding and absence of participation of CYP enzymes, baricitinib may be regarded an appropriate alternative for the treatment of COVID-19 patients.

Numerous studies have been conducted to determine the efficacy of baricitinib in the treatment of COVID-19 patients. In one such trial, the safety and efficacy of baricitinib was evaluated in COVID-19 patients, and it was discovered that 2 weeks of baricitinib treatment resulted in clinical improvements with no adverse effects. This shows that short-term therapy with baricitinib may reduce viral replication and aberrant host inflammation. Another research on animals (Rhesus macaques) found that baricitinib decreased inflammation and repressed inflammation-related cytokine and chemokine activities (Hoang et al., 2021). The findings imply that baricitinib may be one of the optimal potential routes that has been employed as a feasible therapy for SARS-CoV-2-induced inflammation. These data suggest that baricitinib may be a good alternative for decreasing inflammation caused by SARS-CoV-2.

The antiviral efficacy of baricitinib is being examined in multiple ongoing clinical trials, either alone or in combination with other treatments, pharmaceuticals, or antivirals to battle COVID-19. In an adaptive COVID-19 therapy study, baricitinib’s antiviral efficacy against SARS-CoV-2 is being investigated in conjunction with remdesivir (ACTT-2; NCT04401579). The FDA authorized this combination in November 2020 for the treatment of hospitalized COVID-19 patients who need oxygen (FDA-News, 2020a).

### Inhibitors of AXL Kinases

AXL is a tyrosine kinase located on the surface of the cell that controls cell replication, growth, differentiation, and immunity (Ohashi et al., 1995; Stitt et al., 1995; Goruppi et al., 1996; Lu and Lemke, 2001). Activation of AXL by ligands such as vitamin-K dependent GAS-6 (protein growth arrest-specific gene 6) mimics several downstream signaling pathways including RAS/ERK, PI3K-AKT-mTOR, MEK-ERK, NF-B, JAK/STAT, and p38 (Allen et al., 2002; Hafizi and Dahlbäck, 2006; Gay et al., 2017). During the Zika virus infection, AXL kinase played a critical role in viral entry, facilitating viral entrance and modulating the antiviral status of human Sertoli cells (Shimojima et al., 2006; Strange et al., 2019). Inhibition of AXL kinase resulted in the suppression of SOCS1 and SOCS2 proteins (cytokine signaling), increased the expression of IFN, and decreased replication. In a recently published article, the authors stated that the interaction between AXL and the SARS-CoV-2 S glycoprotein on the host cell paved the way for the entry of the virus into the host cell. When the AXL gene was knocked down in H1299 and A549 cells, viral entrance into pulmonary and bronchial epithelial cells was reduced, demonstrating that AXL was required for the virus to enter the host cell. Importantly, pharmacological inhibition of AXL also shows promise as a potential COVID-19 therapy, and bemcentinib is currently being tested for the treatment of patients with COVID-19. Furthermore, gilteritinib has been shown to have antiviral activity against SARS-CoV-2 (Zdżalik-Bielecka et al., 2021). potent inhibitor of AXL kinase, gilteritinib (Lee et al., 2017), is one of the 24 FDA-approved drugs that shows activity against SAR-CoV-2 *in vitro* in a currently unpublished article which is in preprint (0.1 M IC50 10 M; Jeon et al., 2020). It was shown to inhibit SARS-CoV-2 replication in a cellular assay with an IC_50_ of 0.807 M (Bouhaddou et al., 2020). Gilteritinib is an approved drug to treat adult patients suffering from the mutant FLT3-positive refractory or relapsed AML.

### Inhibitors of Epidermal Growth Factor Receptor (EGFR)

EGFR is a tyrosine kinase receptor and its activation effects most of the cell processes, such as growth, development, survival, and cell proliferation (Yarden, 2001). It is also one of the important pathways in the pathogenesis of cancer and is also associated with the infection of various viruses (Zhu et al., 2009; Kung et al., 2011; Beerli et al., 2019), like the spread and mortality of the vaccinia virus, the endocytosis events in influenza A and HCV viruses, and the process of entry for EpsteinBarr viruses (Eierhoff et al., 2010; Lupberger et al., 2011).

Osimertinib, an efficient inhibitor of EGFR, is one of the 24 FDA-approved drugs that have shown anti-SARS-CoV-2 activity in *in vitro* studies (Jeon et al., 2020). Osimertinib effectively inhibited the S protein in SARS-CoV-2 with an EC50 of 3.98 M. Further, it rescued 60% of the SARS-CoV-2 cytopathogenic effect, although the therapeutic window was limited due to its cytotoxic effect (Chen et al., 2020). It is a medication that has been approved for the treatment of non-small cell lung cancer (Ramalingam et al., 2020).

### Inhibitors of PI3K/Akt/mTOR Pathway

In the cell, the GFRs regulate and incorporate various signaling processes. To control proliferation, it incorporates protein kinase B (AKT) into mTORCI signaling via phosphatidyl-inositol 3-kinase [PI3K]. Through their study, Klann and his coworkers concluded that the replication of SARS-CoV-2 can be inhibited by EGFR inhibitors (Klann et al., 2020). In their *in-vitro* study, they used the virus-permissive colon epithelial cell line Caco-2 to create a SARSCoV-2 infected cell culture (Ren et al., 2006; Herzog et al., 2008). A wide range of rearrangements in the signaling pathway, especially with respect to GFR, was observed after the SARS-CoV-2 infection.

Pictilisib is a potent inhibitor of PI3K/-inhibited replication in cells with an IC50 of 2.58 M, and omipalisib, a dual PI3K/mTOR inhibitor, inhibited replication with an IC_50_ of 0.014 M (Ren et al., 2006). Both sorafenib and RO5126766, dual RAK/MEK inhibitors, inhibited viral replication; both these compounds blocked the cytopathic effect during viral infection and replication.

Another drug that inhibited the SARS-CoV-2 viral replication with an IC_50_ value of 4.99 M is the RAS inhibitor lonafarnib (Dhillon, 2021). The antiviral activity of these drugs in SARS-CoV-2 infected UKF-RC-2 cells was within the clinical concentration (Eskens et al., 2001; Martinez-Garcia et al., 2012; Fucile et al., 2015; Sarker et al., 2015; Munster et al., 2016; Perez-Riverol et al., 2019).

### Cyclin Dependent Kinase Inhibitors or Inhibitors of CDK

Cyclin dependent kinase plays a vital role in the progression of the cell cycle as many of the anticancer drugs target the CDKs. CDK has also been the target of various infectious diseases where its expression and function are altered by viruses in the host cell (Klann et al., 2020), such as HIV, Herpes simplex virus (HSV), Zika virus, and hepatitis B virus (HBV) (Schang et al., 2006). Recent literature sheds light on the evidence of CK2’s involvement in the mechanism of down-regulating the ability of the cell to generate IFN–in response to viral infection (DiNicolantonio and McCarty, 2020). Hence, intervention involving CK2 may be considered as one of the important strategies in antiviral treatment. In the infection of vaccinia, CK2 is known to mediate the formation of actin tails that enable the movement of a virus from cell to cell more efficiently (Alvarez and Agaisse, 2012). The spread of the vaccinia virus and the formation of actin tails were impeded by the inhibition of CK2 by the selective inhibitor TBBz at 8.0 M. According to a recent analysis, it was shown that in the case of SARS-CoV-2, CK2 was directly targeted by the nucleocapsid and colocalized at the filopodia protrusions, which may be important for the rapid spread and movement of a virus from cell to cell (Bouhaddou et al., 2020).

Jeon et al. (2020) have reported *in-vitro* anti-SARS-CoV-2 activity of the CDK inhibitor abemaciclib (0.1 M IC50 10 M), which is recognized as one of the 24 FDA-approved drugs. It is a licensed drug used in the treatment of advanced or metastatic breast cancer.

Silmitasertib exhibited significant antiviral activity with an IC_50_ of 1.28 M, suggesting that it may have a prominent role in the SARS-CoV-2 life cycle. A phase II clinical trial is under way on the use of silmitasertib in COVID-19 management (Pillaiyar and Laufer, 2021).

In another clinical study, the role of CIGB-325 (formerly known as CIGB-300) on CK2 for the infection of coronavirus was investigated (Cruz et al., 2021). Twenty patients were selected randomly, and ten COVID-19 patients with pneumonia on the seventh day were given CIGB-325 (2.5 mg/kg for 5 days) along with standard care, while the other ten were given only standard care. Chest computed tomography was enhanced in patients with CIGB-325 and standard care. This suggests that inhibiting CK2 may be one of the best options for treating COVID-19 patients.

To choose CK2 inhibitors as one of the potential therapies for COVID-19 treatment, a large number of inhibitors must be studied. Some of the CK2 inhibitors that may be considered or that are being suggested as potential candidates for COVID-19 infection include quercetin and enzymatically modified isoquercitrin (EMIQ) (Colunga Biancatelli et al., 2020; DiNicolantonio and McCarty, 2020).

### Kinase Inhibitors as Possible Therapeutic Agent to Overcome Respiratory Distress or Complications in COVID-19 Patients

Approved kinase inhibitors, which are used to treat a variety of cancers, include anti-inflammatory and cytokine inhibitory properties, which may help to lower the severity of life-threatening problems caused by the lung damage caused by the respiratory viral infection. Proteins that are associated with reparatory distress or complications are the targets of small-molecule kinase inhibitors. These proteins that cause respiratory distress and that are the targets of kinase inhibitors include those that contribute to the cytokine release syndrome (cytokines–IL-6 and TNF-α) and proteins associated with inflammation and the induction of pulmonary fibrosis (pro-inflammatory cytokine TGF-β).

Environmental stress, pathogenic infection, and pro-inflammatory cytokines are all known to activate the p38 MAPK signaling pathway, which is also involved in cell processes like cell differentiation, apoptosis, and autophagy. Several viruses activate the p38 MAPK signaling pathway, resulting in an unprecedented inflammatory response associated with serious diseases. These viruses that activate the p38 MAPK pathway include SARS-CoV-1, DENV, and IBA. These p38 MAPK signaling pathways could be exploited to limit the generation of proinflammatory cytokines during viral infection. (Fu et al., 2014; Jimenez-Guardeo et al., 2014; Growcott et al., 2018).

The uncontrolled inflammatory impact, which leads to ARDS and myocarditis, is the leading cause of mortality in severe COVID-19 patients (Ruan et al., 2020). In animal models, p38 MAPK has been linked to the injury of the lung and myocarditis (Ma et al., 1999; Fang et al., 2017). Synthesis of cytokines is enhanced when the p38 MAPK pathway is activated (Zarubin and Han, 2005). One of the causative factors for inflammatory injury in COVID-19 patients is suggested to be the up-regulation of the p38 MAPK pathway.

STAT3 is a receptor for the inflammatory cytokine IL-6, which stimulates TGF-1 and may play a role in the development of pulmonary fibrosis in patients suffering from COVID-19 (Matsuyama et al., 2020). As mentioned earlier, in COVID-19 patients the levels of cytokines are elevated, and these cytokines are known to play a key role in the development of ARDS, leading to multiple organ failure and leading to death in severe COVID-19 patients. This shows that inhibiting the cytokine storm via the JAK-STAT signaling pathway could be a promising way to improve current COVID-19 clinical management strategies (Convertino et al., 2020; Ingraham et al., 2020; Luo et al., 2020; Seif et al., 2020). In fact, there are several ongoing clinical trials to study the JAK-STAT signaling pathway inhibitors in patients with COVID-19.

Ivermectin is a drug with a broad spectrum of effects. It is an antiparasitic and antiviral medication. It was found to have antiviral action against a variety of positive-sense ssRNA viruses, including SARSCoV-2. Ivermectin suppressed SARS-CoV-2 in a cellular investigation with an IC_50_ of 2.0 M, making it a potential candidate in the management of COVID-19 in reusing the drug approach (Caly et al., 2020). According to the authors, ivermectin inhibited PNA/KPNB1-mediated viral protein trafficking into the nucleus. Ivermectin, on the other hand, reduces the production of STAT3 and IL-6 by inhibiting p21 activated kinase (PAK1), an oncogenic serine threonine kinase.

Tofacitinib is a JAK inhibitor that can be taken orally and is used to treat RA, which is an autoimmune illness (Berekmeri et al., 2018). With an EC_50_ of 5 nM, tofacitinib inhibits JAK3 and TYK2, reducing inflammatory cytokines associated with RA such as IL-2, IL-4, IL-6, and IL-7. Tofacitinib has a good pharmacokinetic profile, with 74 percent oral bioavailability and a half-life of 2.3–3.1 h (Wollenhaupt et al., 2019). tofacitinib’s safety profile and effectiveness in RA patients were both consistent. The use of tofacitinib, on the other hand, can cause or carry the risk of herpes zoster, cellulitis infections, and thrombosis (Mease et al., 2020).

The efficacy of this medicine was proven in COVID-19 individuals with a 13-year history of ulcerative colitis. According to recent literature, after 2 weeks, all the symptoms were healed without being hospitalized (Jacobs et al., 2020). The study concluded that tofacitinib medication in SARS-CoV-2 patients can be used, although it did not prove that tofacitinib actually helped the COVID-19 patients recover. Several phase II clinical trials are underway to study the efficacy of tofacitinib alone (NCT04412252, phase II), NCT04415151, phase II, NCT04332042, phase II) and in combination with hydroxychloroquine (NCT04390061, phase II).

In another study, fedratinib, a specific JAK2 inhibitor, was found to inhibit the production of TH17-related cytokines, suggesting that it could be beneficial for SARS-CoV-2 patients experiencing a cytokine storm (Wu and Yang, 2020).

Ruxolitinib is an orally accessible medicine (JAK inhibitor) that reduces the generation of cytokines in myelofibrosis, polycythemia vera, and acute graft-versus-host disease patients. Due to its efficiency in treating diseases that are linked to hyper-immune syndrome, the efficacy of ruxolitinib has been tested in patients with COVID-19. In the study, the patients who were given ruxolitinib and standard care had faster clinical recovery and fewer severe adverse events in comparison to the control group. Currently, around 20 clinical trials are underway to study the efficacy of ruxolitinib on COVID-19 patients (Gavegnano et al., 2014; Cao Y. et al., 2020). Some of the studies have been reported to show that it has the ability to help patients overcome ARDS (La Rosée et al., 2020).

One of the most common side effects associated with medication of ruxolitinib (Strand et al., 2015) and baricitinib (Mead et al., 2015) in patients is urinary tract infection and upper respiratory tract infection. The inhibitors of JAK are also known to have a high risk of viral infection, such as herpes virus infection. In a recent study, 2 of the COVID-19 patients with ruxolitinib were reported to experience hematologic toxicity. One of the patients developed infection in soft tissue and the other developed infection linked to herpes labialis. These two cases of infection resulted in the termination of the medication for these two patients.

According to a recent assessment, SARS-CoV-2 infection resulted in minimal IFN expression but significantly increased chemotactic and inflammatory responses, including CCL2, CCL8, IL-6, IL-1, and IL1RA. Infection with SARS-CoV-2 prevents IFN-mediated immune cell recruitment and promotes IFN-stimulated genes in patients. The receptor for the entry of SARS-CoV-2 is a protein that is found on the surface of the virus. ACE is an interferon-stimulated gene (ISG) that is expressed mostly in the airway epithelial cells in humans (Blanco-Melo et al., 2020).

## FDA Approved Therapeutics In the Management of COVID-19

These findings imply that further study is needed to identify the function of IFNs in the response to COVID-19 infection, but inhibition of the JAK pathway may potentially be used to limit SARS-CoV-2 infection. Selective JAK2 inhibition (for example, fedratinib) or combined JAK2 and JAK3 inhibition (for example, tofacitinib) may also be advantageous since they do not impair JAK-type-1-mediated antiviral and antibacterial immunity (Roskoski, 2016; Ziegler et al., 2020).

Remdesivir was the first US-FDA approved drug to be used in hospitalized COVID-19 patients (Rizk et al., 2021). Favipiravir was approved as a repurposed agent for the management of COVID-19 (Naik and Shakya, 2020; Alam et al., 2021). The US-FDA approved different monoclonal antibodies as Emergency Use Authorization (EUA) for bamlanivimab (Rizk et al., 2021) and tocilizumab (Younis et al., 2021). These are recombinant neutralizing human IgG1 monoclonal antibodies. Casirivimab and imdevimab (REGEN-COV) are human immunoglobulin G-1 (IgG1) monoclonal antibodies developed using recombinant DNA technology (Rizk et al., 2021). The monoclonal antibodies are explicitly directed against the spike protein of SARS-CoV-2, preventing the virus from attaching to and entering human cells.

## Conclusion

Given that researchers believe the SARS-CoV-2 virus will persist for an extended length of time, one of the therapy options remaining to halt the virus’s spread is to repurpose current antiviral or medications. Numerous considerations must be addressed when repurposing an authorized pharmaceutical for a new therapeutic use, one of which is identifying efficient inhibitors of critical protein targets. This element acts as a viable therapeutic alternative in this instance. Numerous antiviral medicines are being investigated in clinical trials to treat COVID-19 patients, either alone or in conjunction with other treatments. Numerous kinase inhibitors exhibit antiviral activity because they specifically target important virus–associated proteins and proteins implicated in the formation or progression of SARS-CoV-2 symptoms such as pneumonia, ARDS, cytokine storm, systemic inflammation, and fibrosis. The majority of these kinase inhibitors that have showed antiviral activity in prior viral infections are in various clinical trial stages I through III. To repurpose a medicine effectively, one must first evaluate the substance’s or therapeutic agent’s pharmacokinetics. Because there is an immediate need to treat COVID-19 patients’ symptoms, medications that need prolonged administration to achieve an acceptable therapeutic dosage and anti-inflammatory impact may be difficult to cure or lessen COVID-19 patients’ symptoms. These medications may not be a feasible treatment choice for COVID-19 patients. Similarly, the adverse effects of different kinase inhibitors must be considered, as they complicate the management of SARS-CoV-2 patients. On the other hand, a short-term dosage may mitigate these undesirable effects.

Other, less-explored options for COVID-19 treatment may be examined, such as combination therapy, which may have favorable results. These medicines have already been investigated and found to be beneficial in preventing or treating life-threatening infections such as HIV. Combination strategies may include kinase inhibitors that specifically target the protein, proteins associated with viruses, and proteins related with lung health, or a combination of antivirals and kinase inhibitors (ritonavir-lopinavir, remdesivir, sunitinib, sorafenib, imatinib, gilteritinib, osimertinib, etc.). Another possibility for battling the COVID-19 pandemic is to attack the JAK-STAT signaling system. As previously stated, JAK-STAT promotes hyper-immune activation in SARS-CoV-2 patients via pro-inflammatory cytokines, resulting in ARDS, multiple organ failure, and death. As a result, an approach involving the repurposing of FDA-approved and experimental drugs that target the JAK-STAT signaling pathway may be beneficial.
