# Supplementary material for: Kinase Inhibitors as Potential Therapeutic Agents in the Treatment of COVID-19
Source: Front Pharmacol. 2022 Apr 4;13:806568. doi: 10.3389/fphar.2022.806568 (PMC9014181; doi:10.3389/fphar.2022.806568)
Supplement: Supplementary file 1 [file Table1.docx]

**Supplementary Table 1. Tyrosine kinase inhibitor and their mechanism of action and therapeutic uses**

| **SN** | **Drug  (alphabetical)** | **Structure** | **Mechanism of action** | **Therapeutic uses and drug targets** | **Rationale for use in COVID-19** | **Reference** |
| --- | --- | --- | --- | --- | --- | --- |
| 1 | Abemaciclib |  | CDK inhibitor,  inhibits CDK4 and 6 -inhibits Rb phosphorylation resulting in a G1 arrest and inhibition of proliferation, | FDA approved drug for advanced / metastatic breast cancer.  **Targets:** A specific CDK4/6 inhibitor | antiviral MOA of CDK inhibitors is blocking viral genome replication in host cell | (Corona and Generali, 2018; Jeon et al., 2020); |
| 2 | Baricitinib |  | JAK inhibitor/AAKI kinase and binds to GAK selectively and reversibly inhibits JAK1 and JAK2 to modulates their signaling pathways - reducing the phosphorylation and activation of STATs | Used in RA  **Targets:** Tyrosine-protein kinase JAK1, Tyrosine-protein kinase JAK2, TProtein-tyrosine kinase 2-beta, Tyrosine-protein kinase JAK3 | Decreased ARDS and interfered with the entry and assembly of SARS-CoV-2 into target cell through inhibition of AAKI signals | (Mead et al., 2015; Rodriguez-Garcia et al., 2021) |
| 3 | CIGB-325 | 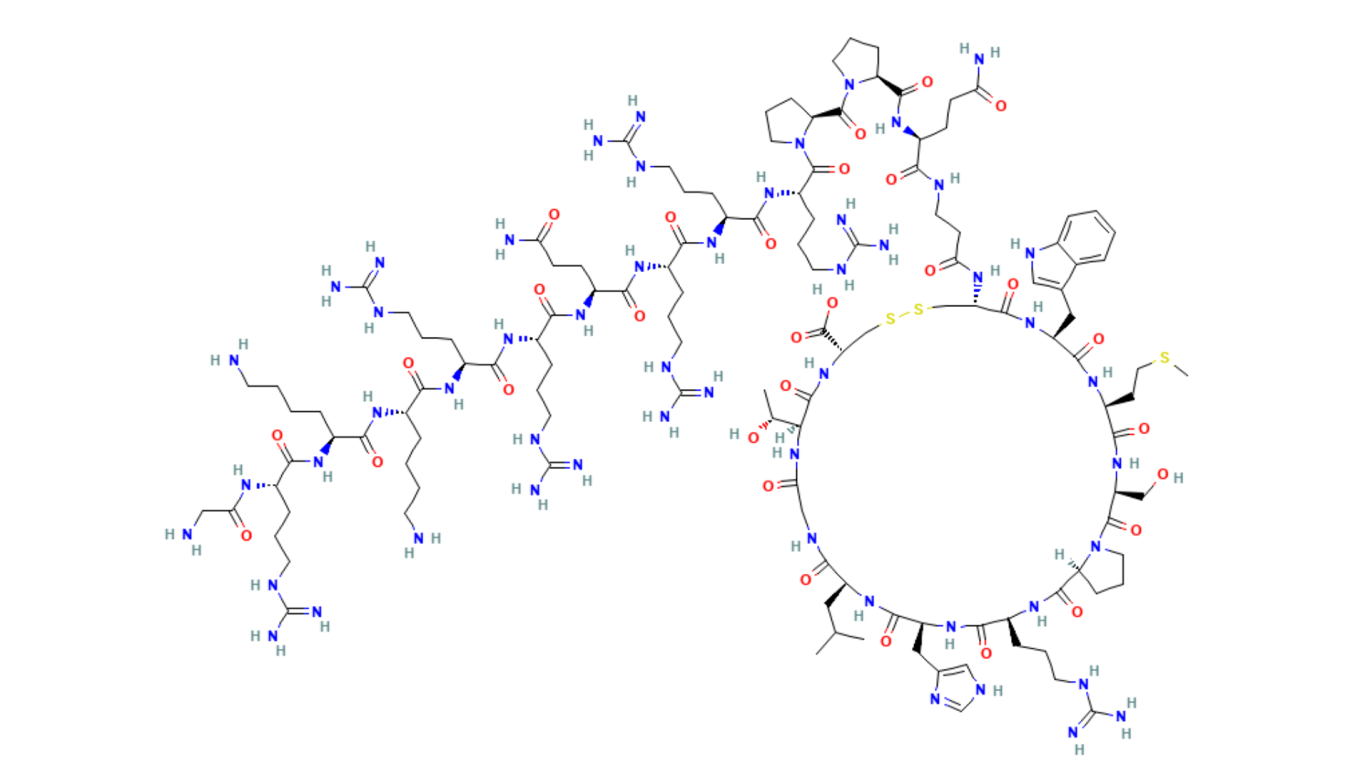 | Inhibit CK2 | A synthetic peptide designed to target a subset of**CK2 91 substrates** by binding to the conserved phosphoacceptor sites and recently it has shown a direct 92 impact over the CK2 enzymatic activity as classical inhibitors | CK2 is involved in downregulation of cell ability to generate IFN-1 in response to viral infection | (Perera et al. 2020, Cruz et al., 2021,) |
| 4 | Dasatinib |  | [SRC-family protein-tyrosine kinases inhibitor](https://pubchem.ncbi.nlm.nih.gov/compound/tyrosine) | Treatment of CML and Acute Lymphoblastic leukemia (ALL)  **Targets:** BCRABL, SRC, Ephrins and GFR. | Antiviral activity contributed to inhibition SFKs like c-Src & Fyn, Effective against dengue virus, and targeted Fyn for RNA  replication | https://pubchem.ncbi.nlm.nih.gov/compound/Dasatinib |
| 5 | Erlotinib |  | EGFR Inhibitor/ Tyrosine Kinase inhibitor  Erlotinib inhibits the intracellular phosphorylation of tyrosine kinase associated with the epidermal growth factor receptor (EGFR) | FDA approved Drug for metastatic Non-small Cell Lung Cancer (NSCLC) and pancreatic cancer  **Target:** Nuclear receptor subfamily1 group I member 2- agonist | Suppressed AAK1 and GAK and exhibited antiviral activity | (Sarah, 2017; Richardson et al., 2020) |
| 6 | Fedratinib |  | JAK-2 Inhibitor  inhibition of JAK2 inhibits phosphorylation of signal transducer and activator of transcription (STAT) 3 and 5, which prevents cell division and induces apoptosis | **Targets**: Tyrosine-protein kinase JAK2, Receptor-type tyrosine-protein kinase FLT3, Tyrosine-protein kinase JAK1 as inhibitor | The cytokines level is elevated during COVID-19 infection, play crucial role in ARDS, multiple organ failure and death, inhibiting cytokine storm through AK-STAT may be beneficial | (Wu and Yang, 2020) |
| 7 | Gemcitabine |  | SRC kinase Inhibitor  inhibition of DNA synthesis  When dFdCTP is incorporated into DNA, a single deoxynucleotide is incorporated afterwards, preventing chain elongation | Testicular cancer, breast cancer, ovarian cancer, non-small cell lung cancer, pancreatic cancer, and bladder cancer  **Targets:** DNA - cross-linking/ alkylation, Ribonuleoside – diphaspate reductase large subunit – inhibitor, UMP- CM - inhibitor | Synergistic effect with Saracatinib | (Singh, et al.2015) |
| 8 | Gilteritinib |  | FLT-3 Inhibitor  selective inhibitor of both- mutations, internal tandem duplication (ITD) and tyrosine kinase domain (TKD), of the FLT3 receptor. Inhibits FLT3 receptor signaling and proliferation | Treatment for Patients Suffering from Mutant FLT-3 positive refractory /relapsed AML  **Targets**  Receptor type tyrosine protein kinase – FLT3, Serotonin receptor, Tyrosine protein kinase receptor, Alk tyrosine kinase receptor | Anti SARS-CoV-2 replication –inhibition of AXL kinase upstream of p38 | (Lee et al., 2017; Zdżalik-Bielecka et al., 2021) |
| 9 | Imatinib |  | ABL inhibitor  inhibits the Bcr-Abl tyrosine kinase, inhibits the receptor tyrosine kinases for platelet derived growth factor (PDGF) and stem cell factor (SCF) - called c-kit. | Chronic Myeloid Leukemia (CML)  **Targets:** Breakpoint cluster region protein  Mast/stem cell growth factor receptor kit, RET poto -oncogene | SARS CoV-1 and MERS-CoV rely on ABL2 kinase for entry imatinib inhibits ABL2 kinase and block viral entry | (Coleman et al., 2016; Budak et al., 2021) |
| 10 | Ivermectin |  | PNA/ KPNB  IVM targets the IMPα component of the IMP α/β1 heterodimer and binds to it, preventing interaction with IMP β1, subsequently blocking the nuclear transport of viral proteins | Broad spectrum anti-parasitic  **Targets:** It kills parasites by activating glutamate-gated Cl- channels, and it also targets several ligand-gated ion channels and receptors, including Cys-loop receptors, P2X4 receptors and fernesoid X receptors. IVM also activates a novel target, the G-protein-gated inwardly rectifying K+ channels | 1. inhibiting the host importin alpha/beta-1 nuclear transport proteins.  2.  may interfere with the attachment of the SARS-CoV-2 spike protein to the human cell membrane | (Chen and Kubo, 2018; Caly et al., 2020; Lehrer and Rheinstein, 2020; Yang et al., 2020) |
| 11 | Lonafarnib |  | RAK/MEKT inhibitor  inhibits farnesyltransferase enzyme  Inhibition of progerin farnesylation reduces progerin accumulation in the inner nuclear membrane, which subsequently slows the progression of HGPS and other progeroid laminopathies | Hepatitis D virus (HDV) infections, and progeria and progeroid laminopathies  **Targets:** [Protein farnesyl transferase/ geranyl geranyl transferase type-1 subunit alpha](https://go.drugbank.com/drugs/DB06448#BE0002373) [Protein farnesyl transferase subunit beta](https://go.drugbank.com/drugs/DB06448#BE0002372) | Inhibited replication of SARS-CoV-2 in UKF-RC-2 cells infected with SARS-CoV-2 | (Dhillon, 2021; Pillaiyar and Laufer, 2021) |
| 12 | Nilotinib |  | ABL inhibitor  inhibits the tyrosine kinase activity of the BCR-ABL protein. Nilotinib fits into the ATP-binding site of the BCR-ABL | Chronic Myeloid Leukemia  **Targets:**  [Tyrosine-protein kinase ABL1](https://go.drugbank.com/drugs/DB04868#BE0000014) [Mast/stem cell growth factor receptor Kit](https://go.drugbank.com/drugs/DB04868#BE0000453) | Nilotinib inhibits SARS-CoV | (Coleman et al., 2016; Sisk et al., 2018) |
| 13 | Omipalisib |  | Inhibitor of PI3K/mTOR | Pathogenesis of idiopathic pulmonary fibrosis  **Targets:** a potent inhibitor of PI3K/mTOR. | Slows the fibrosis development in patients with idiopathic pulmonary fibrosis & coronavirus patients may be related to deregulation of signaling pathway of GFR | (Ren et al., 2006; Pillaiyar and Laufer, 2021) |
| 14 | Osimertinib |  | EGFR inhibitor,   epidermal growth factor receptor (EGFR) tyrosine kinase inhibitor (TKI) that binds to certain mutant forms of EGFR (T790M, L858R, and exon 19 deletion) that predominate in non-small cell lung cancer (NSCLC) tumours | FDA approved drug for Non-Small Cell Lung Cancer  **Targets:** Epidermal growth factor receptor | EGFR is linked to entry of various viruses and targetting this would be an viable option  inhibitor of SARS-CoV-2 S protein | (Ramalingam et al., 2020) |
| 15 | Pictilisib |  | Inhibitor of PI3K/AKTmTOR pathway/ Inhibitor of PI3Kα/δ inhibitor  inhibitor of class I phosphatidylinositol 3 kinase (PI3K), with potential antineoplastic activity, pictilisib selectively binds to PI3K in an [ATP](https://pubchem.ncbi.nlm.nih.gov/compound/ATP)-competitive manner, inhibiting the production of the secondary messenger [phosphatidylinositol-3,4,5-trisphosphate](https://pubchem.ncbi.nlm.nih.gov/compound/phosphatidylinositol-3%2C4%2C5-trisphosphate) ([PIP3](https://pubchem.ncbi.nlm.nih.gov/compound/PIP3)) and activation of the PI3K/Akt signaling pathway | Breast cancer  **Targets**:  [phosphatidylinositol-3,4,5-trisphosphate](https://pubchem.ncbi.nlm.nih.gov/compound/phosphatidylinositol-3%2C4%2C5-trisphosphate) ([PIP3](https://pubchem.ncbi.nlm.nih.gov/compound/PIP3)) | Slows the fibrosis development in patients with idiopathic pulmonary fibrosis & coronavirus patients may be related to deregulation of signaling pathway of GFR | (Sarker et al., 2015) |
| 16 | RO5126766 |  | RAK/MEKT inhibitor | Solid Tumors  **Targets:** a dual RAF/MEK inhibitor | Block viral replication, block cytopathic effect during viral infection and replication | (Martinez-Garcia et al., 2012) |
| 17 | Ruxolitinib |  | JAK1 and JAK2 inhibitor, reduces cytokines  Ruxolitinib is a selective and potent inhibitor of JAK2 and JAK1, with some affinity against JAK3 and TYK2. Anticancer effects of ruxolitinib are attributed to its inhibition of JAKs and JAK-mediated phosphorylation of STAT3. Inhibits myeloproliferation and suppresses the plasma levels of pro-inflammatory cytokines such as IL-6 and TNF-α | Treatment of myelofibrosis  **Targets:**  Tyrosine protein kinase JAK 2, JAK 1, JAK 3 and Non -receptor Tyrosine protein kinase TYK 2 | The cytokines level is elevated duringCOVID-19infection, play crucial role in ARDS, multiple organ failure and death, inhibiting cytokine storm through AK-STAT may be beneficial  . | (Martinez-Garcia et al., 2012; Jianguo et al., 2020; La Rosée et al., 2020) |
| 18 | Silmitasertib |  | CDK inhibitor, | Recurrent sonic hedgehog (SHH) driven medullo-blastoma,  Targets: targets the CK2 pathway, showed efficacy in Covid-19 | Silmitasertib ,  CK2 is involved in downregulation of cell ability to generate IFN-1 in response to viral infection | (Silva-Pavez et al., 2019; Pillaiyar and Laufer, 2021) |
| 19 | Saracatinib |  | Inhibitor of SRC kinase /ABL kinase/ SFK ignaling pathway  dual-specific inhibitor of Src and Abl, protein tyrosine kinases | Treatment of idiopathic pulmonary fibrosis (IPF)  **Targets:** inhibitor of the [protein tyrosine kinase Fyn](https://www.sciencedirect.com/topics/pharmacology-toxicology-and-pharmaceutical-science/protein-kinase-fyn), | Antiviral activity contributed to inihibition SFKs like c-Src & Fyn  Effective against  dengue virus, and targeted Fyn for RNA  replication | <https://pubchem.ncbi.nlm.nih.gov/compound/Saracatinib>  (Abuserewa et al., 2021) |
| 20 | Sorafenib |  | RAK/MEKT inhibitor  target the Raf/Mek/Erk pathway. By inhibiting these kinases, genetic transcription involving cell proliferation and angiogenesis is inhibited | Treat kidney, liver, and thyroid cancer.  **Targets :**[Serine/threonine-protein kinase B-raf](https://go.drugbank.com/drugs/DB00398#BE0000634) – inhibitor, [RAF proto-oncogene serine/threonine-protein kinase](https://go.drugbank.com/drugs/DB00398#BE0000036) – as inhibitor  [Vascular endothelial growth factor receptor 3](https://go.drugbank.com/drugs/DB00398#BE0000023)- antagonist  [Vascular endothelial growth factor receptor 2](https://go.drugbank.com/drugs/DB00398#BE0000369) - antagonist | Block viral replication, block cytopathic effect during viral infection and replication | (Fucile et al., 2015) |
| 21 | Sunitinib |  | Multi Target kinases  inhibits multiple RTKs  platelet-derived growth factor receptors (PDGFRa and PDGFRb), vascular endothelial growth factor receptors (VEGFR1, VEGFR2 and VEGFR3), stem cell factor receptor (KIT), Fms-like tyrosine kinase-3 (FLT3), colony stimulating factor receptor Type 1 (CSF-1R), and the glial cell-line derived neurotrophic factor receptor (RET) | Renal Cell Carcinoma/Imitanib resistant gastrointestinal stromal tumor  **Targets :**[Platelet-derived growth factor receptor beta](https://go.drugbank.com/drugs/DB01268#BE0000205)  v[ascular endothelial growth factor receptor 1](https://go.drugbank.com/drugs/DB01268#BE0000029), [Mast/ stem cell growth factor receptor Kit](https://go.drugbank.com/drugs/DB01268#BE0000453), [Vascular endothelial growth factor receptor 2](https://go.drugbank.com/drugs/DB01268#BE0000369) | Suppressed AAK1 and GAK and exhibited antiviral activity | (Kelly et al., 2021) |
| 22 | Tofacitinib |  | JAK inhibitor  prevents the phosphorylation and activation of STATs  works therapeutically by inhibiting the JAK-STAT pathway to decrease the inflammatory response | Treatment of psoriasis and psoriatic arthritis  **Targets:**  Tyrosine protein kinase JAK 2, JAK 1, JAK 3 and Non-receptor Tyrosine protein kinase TYK 2 | The cytokines level is elevated duringCOVID-19infection, play crucial role in ARDS, multiple organ failure and death, inhibiting cytokine storm through AK-STAT may be beneficial | (Berekmeri et al., 2018; Varyani et al., 2019) |
